# Q&A with Editorial Board Member Dr David Nelson

**DOI:** 10.1038/s42004-023-00941-2

**Published:** 2023-07-05

## Abstract

Dr David Nelson talks about his motivation for being a chemist, his viewpoints on the research field of homogeneous catalysis, as well as his experience of being an Editorial Board Member for *Communications Chemistry*.

David studied chemistry at the University of Edinburgh (MChem 2008) before moving to the University of Strathclyde to complete his PhD (2012) with Professor Jonathan Percy in the area of alkene metathesis catalysed by ruthenium complexes. He then worked as a Research Fellow with Professor Steven Nolan FRSE at the University of St Andrews on a range of topics across late transition metal chemistry and catalysis. He started his independent career at the University of Strathclyde in 2014 as a Chancellor’s Fellow and was promoted to Senior Lecturer in 2018. His team focuses on understanding transition metal-catalysed reactions, particularly nickel-catalysed reactions and C–H activation reactions. David was awarded a 2020 Thieme Chemistry Journals Prize and the 2021 RSC Inorganic Reaction Mechanisms Early Career Award.

**Figure:**
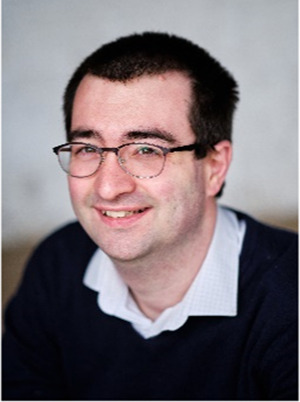
© 2020 Suzanne Black Photography

Why did you choose to be a scientist?

I’ve always been curious about how things work, and so I was always attracted to science and computing subjects when I was at high school. I’d originally considered going into medicine but found that chemistry was really where my passion was, so I pursued a chemistry degree. During my degree, it was really the research side of things that excited me and so I’ve been doing chemistry research ever since!

What scientific development are you currently most excited about?

I’m a bit of a cynic when it comes to ‘hot topics’ – there are some concepts and tools that are emerging now that could really be transformative but sometimes distinguishing true progress from buzzwords and bandwagons is not straightforward. I really enjoy reading work where new reactivity is discovered, established doctrine is challenged, and new ways of thinking about chemistry are introduced. So, I like seeing what’s going on in fields such as catalysis with main group elements, and I really like the fact that more and more teams are really keen to delve into reaction mechanisms and truly understand their chemistry. One of my team members has been collaborating with Jolene Reid (UBC) who has some really nice ways of thinking about how general a catalyst is, as opposed to being fixated purely on the yield in a specific reaction.

What direction do you think your research field should go in?

I think the nickel catalysis field needs to get to grips with the very wide range of reaction mechanisms that can be invoked: there’s no one true mechanism that covers all reactions and the reaction behaviour can be very dependent on ligand and substrate structure. It’s complicated, but if we fully understand what determines the mechanism then there is amazing scope for the fine tuning of synthetic reactions.

What attracted you to becoming an Editorial Board Member for *Communications Chemistry*?

Two things really. Firstly, publishing the outcomes of our work is such an important part of what we do that I think every academic has a responsibility to do their part to make sure that the system is fair and honest and that the technical quality of published papers is as high as possible. Secondly, I’d interacted with the publishing process many times as an author but I’d seen relatively little of what goes on in the background and how the process works. I truly think that seeing how a process works behind the scenes better equips you to interact with that system from the outside. I use the analogy of working in retail. I spent my weekends as an undergraduate student working in DIY and electronics stores and so this influences the way I behave in shops now – I make a point of treating shop staff with respect and I hate seeing things abandoned on the wrong shelves in a supermarket!

What have you gotten out of the experience of being an Editorial Board Member for *Communications Chemistry*?

I’ve learnt a lot about the publishing process, and also about how to write a good review. I’ve read loads of reviews by this point and know which ones are going to help me make a decision on a manuscript and which are not particularly useful. I’ve also been exposed to a lot more varied science than I perhaps would have been if all of my reading was limited to my immediate area of interest. I do get a lot of satisfaction out of seeing a paper come through the process and end up as a final published article that has been improved as a result of constructive feedback from the referees.

How do your editorial responsibilities integrate with your academic role?

Publishing and reviewing research is a big part of an academic job, so I feel that it’s helped me to look at my approach to these activities with a fresh pair of eyes, especially when it comes to writing a review that the editor can confidently make a decision based on. I frequently read and feed back on writing from my team and my colleagues (grants, papers, etc.) so things I’ve learned and examples I’ve seen during my editorial role have helped me here.

What do you see as the role of *Communications Chemistry* in the scientific community?

At the end of the day, I think any journal has to focus on publishing papers of a high technical quality. Assessing ‘impact’ is very subjective, but a journal that covers the whole gamut of chemistry needs to be publishing work that is of some interest to more than one chemistry subdiscipline. General chemistry journals are also a good way to expose readers to a wider range of chemistry than you might otherwise go looking for: you might be browsing an issue to see what relevant papers have come up and something in a quite different area might catch your eye! The journal has also been quite proactive in thinking about data quality and how data are reported – the policies on different types of data are always being refined and are excellent guidelines for any researcher to ensure that their data and its reporting are top notch.

*This interview was conducted by the editors of Communications Chemistry*.

